# Automated Channel Selection in High-Density sEMG for Improved Force Estimation

**DOI:** 10.3390/s20174858

**Published:** 2020-08-27

**Authors:** Gelareh Hajian, Ali Etemad, Evelyn Morin

**Affiliations:** Department of Electrical and Computer Engineering, Queen’s University, Kingston, ON K7L 3N6, Canada; ali.etemad@queensu.ca (A.E.); evelyn.morin@queensu.ca (E.M.)

**Keywords:** high-density electromyography, force estimation, channel selection, fast orthogonal search

## Abstract

Accurate and real-time estimation of force from surface electromyogram (EMG) signals enables a variety of applications. We developed and validated new approaches for selecting subsets of high-density (HD) EMG channels for improved and lower-dimensionality force estimation. First, a large dataset was recorded from a number of participants performing isometric contractions in different postures, while simultaneously recording HD-EMG channels and ground-truth force. The EMG signals were acquired from three linear surface electrode arrays, each with eight monopolar channels, and were placed on the long head and short head of the biceps brachii and brachioradialis. After data collection and pre-processing, fast orthogonal search (FOS) was employed for force estimation. To select a subset of channels, principal component analysis (PCA) in the frequency domain and a novel index called the power-correlation ratio (PCR), which maximizes the spectral power while minimizing similarity to other channels, were used. These approaches were compared to channel selection using time-domain PCA. We selected one, two, and three channels per muscle from the original seven differential channels to reduce the redundancy and correlation in the dataset. In the best case, we achieved an approximate improvement of 30% for force estimation while reducing the dimensionality by 57% for a subset of three channels.

## 1. Introduction

Accurate muscle force estimation enables many applications, including control of powered prostheses, medical rehabilitation, sports medicine, and human–machine interaction [1,2,3,4,5], where electromyogram (EMG) signals have been extensively used. EMG signals can be acquired by electrodes located on the skin surface (non-invasive) or inserted into the muscle tissue (invasive) based on the EMG application. Surface electrodes are affixed to the skin and make contact through an electrolyte that can be either a gel or paste, or sweat in the case of dry electrodes. Surface electrodes are easy to use and provide information about muscle activity, with minimum discomfort to the participant. Invasive needle or wire electrodes are usually used to study the motor unit (MU) activities, since they provide localized information about neuromuscular activity [6]. However, using multi-channel surface EMG electrodes along with signal decomposition algorithms, information at the MU level can be extracted [7]. Therefore, because of the risk of injury, discomfort, and infection that invasive electrodes pose, non-invasive methods are preferable. In addition, for applications like EMG-based muscle force estimation, although invasive methods can be used, this is generally not acceptable in humans due to the mentioned reasons.

The surface EMG (referred to as EMG for simplicity) is often utilized to estimate the underlying neuromuscular activation that leads to force generation. Many studies have been carried out to estimate muscle force by estimating the relationship between EMG signals and output force [8,9,10,11,12,13,14]. Hill’s muscle model [15] is often used, where estimated muscle activation levels are used as inputs to the model, and the generated muscle force is calculated as the output [16,17]. Other methods including polynomial functions [11,12], linear regression [18], neural networks [13,19,20,21], parallel cascade identification [22], and fast orthogonal search (FOS) [8,9,10,16] have been used to identify the EMG–force relation without requiring any knowledge of muscle and joint dynamics.

Conventional EMG–force modeling techniques generally used EMG recorded from a single bipolar electrode attached to the belly of the muscle. Recently, researchers have investigated EMG-based force estimation using multiple bipolar electrode pairs [9,18,22,23] and high-density (HD) EMG recording [10,24,25,26] because of the enhanced information about neuromuscular activation as well as the improved muscle force estimation results [10,22,24]. However, there are challenges associated with using a large number of electrodes. Processing the acquired HD-EMG requires more time and effort due to the high dimensionality of the data, which might contain redundant and correlated information. Additionally, the complexity of the systems, computational cost, and power consumption can make the HD and multi-channel recording applications limited. Studies have been done to manage dealing with recorded HD-EMG data by using fewer channels through ensemble learning techniques [10] and by applying principal component analysis (PCA) [24]. Channel selection approaches can also be implemented to deal with highly correlated information obtained using HD electrodes and to extract the informative subset of channels that contains discriminatory information to reduce the computational cost and time, and hence reduce power consumption, without significantly compromising the performance.

Use of HD-EMG recording, in which data are obtained from an electrode montage placed over the muscle belly, can improve force estimation accuracy because the muscle activation level is better characterized by the multiple channels of data [10]. Johns et al. [10] utilized HD-EMG signals with an ensemble learning technique coupled with the FOS algorithm for force estimation. Monopolar HD-EMG recordings were obtained from the biceps brachii (48 channels), triceps brachii (64 channels), and brachioradialis (eight channels) during a variable-force isometric contraction exercise. Ensemble learning was used to manage the high dimensionality and correlation in HD-EMG data and to reduce the effect of outliers on HD-EMG force estimation. The authors considered a number of different HD-EMG spatial configurations. The highest accuracy was obtained for configurations in which a higher number of channels was used, while the lowest accuracy was achieved for the single channel configuration [10].

The effect of different bipolar electrode configurations and directions on EMG-based force estimation was investigated using HD-EMG recorded from the triceps brachii during isometric–isotonic extension of the elbow [24]. Force estimation error was expressed as the root mean square difference (RMSD) between the normalized force and the normalized EMG. Different configurations were compared to find the best bipolar orientation. Additionally, PCA was used to manage the highly correlated information obtained from HD-EMG recording. PCA was applied to the EMG signals, then the first few modes were discarded, and the linear envelopes of the remaining modes were summed to estimate the neuromuscular activity. With respect to force estimation, the PCA-based approach showed a substantial improvement over other configurations/directions. Therefore, it was concluded that PCA can be considered as an effective tool for extracting relevant force-related information from a high-density EMG grid, thus improving the quality of EMG-based muscle force estimation [24].

Channel selection and dimensionality reduction have been widely used in classification studies of bio-signals to provide faster processing and improved model performances [27,28,29,30,31,32,33,34]. Geng et al. [27] proposed a novel channel selection approach called multi-class common spatial pattern (MCCSP) for channel selection in EMG pattern-recognition-based movement classification. The proposed algorithm involves calculating a matrix in which one motion class variance is maximized and the sum of the variances of all other classes is minimized. Spatial pattern matrices corresponding to all included classes were then obtained, and were utilized to select an optimal subset of channels from 56-channel HD-EMG recordings. Ong et al. [29] used a PCA-based channel selection method for visually evoked potentials to classify alcoholics versus non-alcoholics. They selected 16 from an initial set of 60 channels based on their contribution to the total variance. Their results suggested that the classification performance experienced a negligible decrease when 16 channels were utilized instead of the initial 64 [29]. PCA has also been used for feature selection and sufficient parallel channel selection from sensor arrays to reduce the dimensionality of the raw data to investigate the performance of a bionic olfactory model to classify two datasets—three classes of wine and five classes of green tea [35]. Guler et al. [36] recorded EMG signals from the biceps brachii and abductor digiti minimi muscles to diagnose and classify neuromuscular disorders. The extracted features from the EMG signals were the fast Fourier transform (FFT) coefficients. Before applying the feature set to the multilayer perceptron (MLP) and support vector machine (SVM) classifiers, PCA was used to reduce the dimensionality. Then, PCA coefficients were applied as inputs to classify the EMG data into three conditions: Normal, neuropathy, and myopathy, where the best classification accuracy of 85.4% was obtained by SVM [36]. Shih et al. [30] used a machine learning-based approach to reduce the average number of electroencephalogram (EEG) channels from 18 to 4.6 for seizure detection. A feature selection algorithm with backward elimination was used for channel selection, while the mean detection accuracy only experienced a slight decrease. Several studies have focused on channel selection in EMG applications [31,32,33]. Martinez et al. [31] recorded HD-EMG signals to estimate grasp force. They examined subsets of 4, 8, and 16 channels, in comparison to the full set of 168 channels, to determine the minimum amount of information needed for grasp force prediction. Three channel reduction methods were used: selecting channels in the center of the grid only (Fix-Ridge); selecting the Fix-Ridge channels with feature selection using elastic nets analysis (Fix-EN); and selecting channels using lasso with EN feature selection (LassoG-EN) in [31]. The Fix-EN was selected as the best method, since there was no statistical difference between methods, and 16 channels proved to have the best trade-off between complexity and performance [31]. Al-Ani et al. [32] recorded multi-channel EEG (64 channels) and EMG (eight channels) data to classify up to four alertness states (engaged, calm, drowsy, and sleep) using EEG, and six different grip and finger movements using EMG. A dynamic channel selection method was proposed to select channels that are more relevant to the given classification task for both signal types. This method was compared with exhaustive search, and it was concluded that while for a small number of channels (e.g., eight channels), an exhaustive search is feasible and yields good results, the approach is not possible for a larger number of channels [32]. A novel variable selection method based on Kullback–Leibler (KL) information to select channels to classify four hand motions using EMG has been explored [33]. The measured signals were considered as probability variables, and their probability density functions were estimated using probabilistic neural network learning based on KL information. The results indicated that the average classification rate using the selected channels is almost the same as using all the channels [33].

The goal of this work is to develop an efficient and effective technique for selecting a subset of available HD-EMG channels for force estimation while minimizing the effect on force estimation accuracy. We propose two different approaches: One is based on PCA in the frequency domain and the other is based on a novel algorithm that calculates and maximizes an index, which is called the power-correlation ratio (PCR). Then, the performance of these two proposed approaches is evaluated and compared to the results of channel selection using PCA in the time domain as a baseline. PCA in the time domain has been used by other studies for channel selection, dimensionality reduction, and feature selection [14,24,29,35,37,38,39]. Preliminary versions of this work have been reported by Hajian et al. [26,40]. This paper extends and unifies those works by defining and assigning the PCR method capable of measuring a specific and informative index for channel selection. Moreover, a larger and extended dataset, which contains three different joint angles and two forearm postures, was recorded and analyzed. Subsets of one, two, and three channels were selected using the proposed techniques of PCR and PCA in the frequency domain. Additionally, further analysis was performed where the effects of using more than one principal component (PC) for channel selection by PCA have been investigated. Finally, the results of the proposed PCR method have been compared to PCA in both the time and frequency domains on the extended dataset.

## 2. Methodology

### 2.1. Experimental Setup

**Figure 1:**
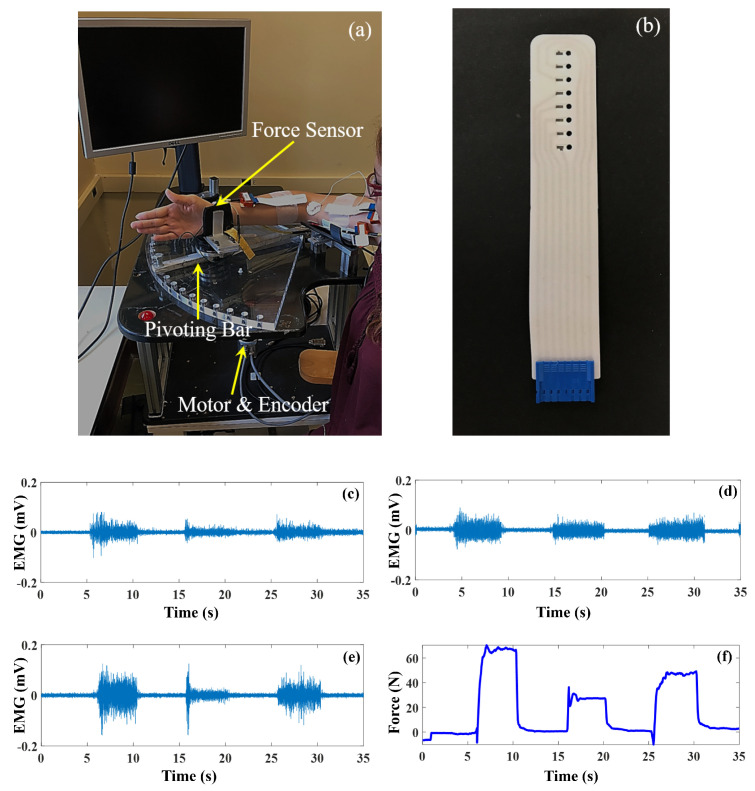
The Queen’s University Arm (QARM) experimental setup and the high-density (HD) electrodes (8 sensors) are presented in (**a**,**b**), respectively. Sample differential electromyograms (EMG) from the long head of biceps brachii (**c**), the short head of biceps brachii (**d**), and the brachioradialis (**e**) are presented. The measured force is also shown (**f**). All records are from one subject, one trial at $90^{\circ }$ elbow joint angle, at neutral posture.

Data collection was performed using the Queen’s University Arm (QARM) [8]. This setup, shown in Figure 1a, is a single-degree-of-freedom (1-DOF) exoskeleton test bed. The right shoulder is abducted 90 degrees and flexed 15 degrees, and the upper arm is fixed in the horizontal plane. The forearm rests on a pivoting aluminum bar such that motion of the arm is constrained to elbow flexion and extension, where the elbow and pivot bar axes of rotation are aligned. The bar can be locked in place for isometric contractions. At the end of the bar is a rigid wrist brace coupled to an ATI 6-DOF Gamma force/torque sensor with a high stiffness of $9.1\times 10^{6}$ N/m, which is used to measure the generated elbow force at the wrist. Force data are sampled at 1000 Hz using a National Instruments data acquisition card. For this study, 13 healthy subjects (5 females and 8 males; age 27±4 years) participated in the experiment. Subjects provided informed consent before starting the experiment. For each subject, the data were collected in a single session. The experimental protocol was approved by the Queen’s University Health Sciences Research Ethics board.

Linear HD-array electrodes (shown in Figure 1b), which have silver/silver chloride (Ag/AgCl) contacts arranged on a flexible plastic substrate, were used. These sensors have 8 monopolar electrodes with a 5 mm inter-electrode distance (IED). The electrodes were attached to the skin using adhesive pads that have small wells filled with conductive paste over the electrode contacts. The electrodes were placed on the right arm of all the participants, where 11 participants were right-hand dominant and 2 were left-handed. The EMG data were collected from three HD-array electrodes, located on the long head and short head of biceps brachii and on the brachioradialis, during isometric elbow flexion for two forearm postures (neutral and supinated). One of the main concerns in EMG signal recording is the location of electrodes, as the electrode placement should be consistent among participants and aligned with the muscle fibers on the belly of the desired muscles. Although some level of variation is unavoidable due to differences among participants, we used the “surface electromyography for the non-invasive assessment of muscles” (SENIAM) sensor location recommendation, which provides recommendations for sensor locations on 30 individual muscles. In SENIAM, the locations are determined based on each subject’s anatomical measurements, which compensates for the differences among subjects. For the long head and short head of biceps brachii, the center of the electrode array (i.e., fourth electrode) was located on the SENIAM sensor location recommendation for the biceps. Sufficient distance between the arrays on the long head and short head of the biceps was insured based on subject’s arm size. For the brachioradialis, the fourth electrode of the array was placed at one-third the length of the forearm measured from the elbow. Standard electrocardiogram (ECG) pre-gelled electrodes with Ag/AgCl contacts were used as reference electrodes, which were placed on regions with lower myoelectric activity. For the brachioradialis, the reference electrode was located on the wrist, while for the long head and short head of the biceps, they were placed on the elbow and fossa cubit (tendon). In addition, two reference electrodes on the right and left wrists were used in a driven-right-leg (DRL) circuit to reduce 60 Hz interference.

The EMG data were collected using the OT Bioelettronica EMG-USB2 HD system. Each EMG signal is hardware band-pass filtered with cut-off frequencies of 10 and 500 Hz, and sampled at 2048 Hz. The experiment was conducted for three elbow joint angles of 60, 90, and 120 degrees at three force levels of 20%, 35%, and 50% of the maximum voluntary contraction (MVC). MVC was measured at the three joint angles and used to generate profiles with three randomly alternating flexion plateaus. In each trial, the subject generated force to follow the force profile at a single joint angle. This was repeated three times at each joint angle for a total of nine trials per forearm posture. The duration of each contraction was 5 s. Appropriate rest periods were provided in order to avoid muscle fatigue. In addition, subjects were instructed not to activate their triceps brachii muscles during flexion. EMG from the triceps brachii muscle was recorded to insure that there was no muscle activation happening during the elbow flexion. This was done in order to minimize the contribution of antagonist muscles to torque about the elbow joint.

### 2.2. Pre-Processing

First, the differential HD-EMG signals were obtained by subtracting neighboring channels, resulting in 7 differential channels from each flexor muscle. Each differential channel was band-pass filtered with cut-off frequencies of 10 and 500 Hz using a fourth-order Butterworth filter. The linear envelopes (LE) of the channels were then obtained by full-wave rectification and smoothing of the EMG signals with a 300-point moving average filter (about 147 ms) to estimate the signal amplitude [9]. Each LE was normalized with respect to the mean of the LE at 50% MVC according to Johns et al. [10]. The force profiles, originally sampled at 1000 Hz, were then up-sampled using linear interpolation to 2048 Hz in order to match the sampling frequency of the EMG. For both the EMG and force data, 3 s of data at each force level of each trial during which the force was constant were extracted for analysis. The normalized EMG LE signals were used for force estimation. Sample differential EMG signals, acquired from the elbow flexor muscles (one channel), and the wrist force data, acquired at $90^{\circ }$ elbow joint angle in a neutral posture, are shown Figure 1c–f.

### 2.3. Force Estimation Using FOS

A nonlinear system identification method, called FOS [41], which estimates the system output as a weighted sum of *M* linear or nonlinear basis functions $p_{m}\left(n\right)$ and coefficient terms $a_{m}$, is shown in Equation (1). y(n) is the measured data, e(n) is the estimation error, and n is the discrete time sample index.

(1) $$y\left(n\right)=\sum_{m=1}^{M}a_{m}p_{m}\left(n\right)+e\left(n\right)$$

FOS aims to minimize the mean square error (MSE) between the estimate and the system output, where the algorithm searches through a large pool of *N* available candidate basis functions (N≫M) in order to select the functions that contribute the most to the reduction of the calculated MSE [16]. This method is based on the principle of Gram–Schmidt orthogonal identification, whereby orthogonal basis functions are generated from the candidate basis functions and coefficients, minimizing the estimated MSE. FOS determines each basis function and corresponding coefficients in a single iteration such that the basis function with the greatest reduction in the estimated error is selected, and this process continues until the stopping criteria are met. Here, the FOS process was stopped when the number of functions reached 9, as suggested by Hashemi et al. [9].

We used one trial of the experiment for each individual subject at each joint angle and forearm posture to train an FOS model and the next two trials to test the model. The inputs to the model consisted of the linear envelopes of the EMG recordings of all channels of the three muscles, as well as the elbow joint angles, while the force measured at the wrist was used as the output. For the basis functions, the set of 57 functions described by Mobasser et al. [8] were employed and computed for each channel of the three muscles. The functions comprise a set of common functions, which include the elbow joint angle (θ), the LEs of each muscle, nd products of sin(θ) and cos(θ) with LEs of individual muscles and with cross-products of LEs of two muscles, and a series of non-linear (quadratic, limited square, square root, and sigmoid) functions.

### 2.4. Proposed Channel Selection Algorithms

In order to select a subset of channels to be used for force estimation with FOS, we aimed to reduce the redundant and common information without considerable compromise to the variance of the recorded EMG data among the different channels. As a result, channels were selected first using PCA, and then using a new method based on maximizing a novel index. The two approaches are described in the following.

#### 2.4.1. PCA-Based Channel Selection

PCA is a technique for decomposing high-dimensional data into a set of linearly independent components called principal components (PC). By projecting the data from a high-dimensional space onto a lower dimensional space, PCA aims to maximize the captured variance within the projected data, thus retaining the maximum amount of information while performing dimensionality reduction [39].

First, PCA in the time domain was applied to the LEs of the 7 differential EMG signals for each muscle, and the subset of channels with the highest coefficients, indicating the highest contribution to the first PC, was selected. Next, the magnitude of the FFT was computed for each of the EMG signals. The phase information was discarded due to its irrelevance, as it corresponds to the delay as the EMG signal travels along the muscle fibers [42]. Then, PCA was applied to the 21 FFT magnitudes, corresponding to 7 channels from each muscle, and the subset of channels per muscle with the greatest contribution to the first PC was selected.

#### 2.4.2. Power-Correlation Ratio Maximization

We developed a new technique for channel selection in EMG-based force estimation. Channels that contain higher force-related information, while having less common information with others, are selected to capture the needed information for EMG-based force modeling, as well as to reduce the redundancy and correlation in the dataset. Thus, to estimate the amount of information in each channel of each muscle, the power spectral densities (PSD) of all channels are calculated, since it has been proven that the PSD of the EMG has significant positive correlation with the generated force [43,44]. Then, the calculated PSDs are normalized against the maximum value over the muscle. This is called the normalized spectral density, which is calculated as:

(2) $$\bar{PSD_{c,m}}=\frac{PSD_{c,m}}{max{\left(PSD\right)}_{m}},$$

where *c* is the evaluated channel, and *m* is the muscle.

While utilizing the channels with the greatest amount of spectral information (after removing the noise components through pre-processing) is required, our goal was to select the subset of channels that would minimize the redundant information. As a result, we used Pearson’s correlation coefficient:

(3) $$r_{x,y}=\frac{\sum_{i=1}^{n}\left(x_{i}-m_{x}\right)\left(y_{i}-m_{y}\right)}{\sqrt{\sum_{i=1}^{n}{\left(x_{i}-m_{x}\right)}^{2}}\sqrt{\sum_{i=1}^{n}{\left(y_{i}-m_{y}\right)}^{2}}},$$

where *r* is the correlation coefficient, *x* and *y* are two time-series of length *n* (e.g., EMG segments, with *n* as a number of samples in each segment), and $m_{x}$ and $m_{y}$ are the mean values of the two time-series.

Next, by averaging the correlation coefficients of each channel against all the other channels, the overall similarity of each channel with the other channels is estimated. This concept is presented in Equation (4), where *c* denotes the given channel and *r* is the set of all the channels excluding *c*.

(4) $$m_{c}=\frac{1}{N-1}\sum_{r=1}^{N}\left|r_{c,r}\right|$$

Finally, PCR for channels of each muscle is calculated as:

(5) $$PCR_{c}=\frac{\bar{PSD_{c}}}{m_{c}}$$

By calculating this index for every channel and selecting the channels with the highest values of PCR, this method ensures that we select those channels with the highest normalized spectral information and minimal correlation with other channels. Channel selection was performed within the 7 individual channels of the HD-electrodes for each of the 3 muscles and 2 postures separately.

### 2.5. Force Modeling

Force modeling was performed by using FOS on: (1) all 7 differential channels per muscle, and (2) subsets of channels derived by applying the proposed channel selection methods (PCA and PCR) on HD channels. The model’s inputs are the linear envelopes of the EMG recordings of all used channels of the three muscles, as well as the elbow joint angles. The ground truth is the recorded force at the wrist.

### 2.6. Model Training and Validation

The PCA and PCR methods were implemented to select a subset of one, two, and three channels per muscle, and the force estimation results were compared with the estimation results of the full set of 7 channels per muscle. The evaluation criterion used was the normalized mean squared error (NMSE) [8,9,10] and is calculated by:

(6) $$\%\mathrm{NMSE}=\frac{\sum_{i=1}^{N}{\left(F_{measured,i}-F_{estimated,i}\right)}^{2}}{\sum_{i=1}^{N}F_{measured,i}^{2}}\times 100,$$

where $F_{measured}$ and $F_{estimated}$ are the measured and estimated wrist forces, respectively, and *N* is the length of the segment.

Force modeling was done in a subject-specific manner so that the data for each subject and each joint angle and forearm posture are used separately to develop a model. For each subject, the first trial of the experiment was used for the FOS model training, and the next two trials were used for testing the model. Averaged test %NMSE values were obtained for each subject. Then, the %NMSE values were obtained by averaging the errors across all subjects for three elbow joint angles and two forearm postures for different channel selection approaches.

### 2.7. Statistical Analysis

Statistical analysis was done using MATLAB (MATLAB 18.1, The MathWorks Inc., Natick, MA, USA). Analysis of variance (ANOVA) and Welch’s t-test were used to determine the statistical significance of the proposed channel selection methods. The independent variable was the %NMSE, and the significance level was set at 5%.

## 3. Results and Discussion

The mean and standard errors (SE) of the %NMSE values for seven channels per muscle and subsets of one, two, and three channels per muscle are presented in Table 1. The channel subsets were obtained using time-domain PCA (PCA${}_{time}$), frequency-domain PCA (PCA${}_{freq}$), and PCR on the HD-EMG data; force estimation was done using FOS. As the number of selected channels increases, the %NMSE values decrease in most cases for different joint angles and forearm postures.

A two-way ANOVA was performed on the results of force estimation with the selected channel subsets and channel selection methods as factors to analyze the significance of changes in force estimation accuracy. ANOVA is a robust method that can be used to show significance even in data with a non-normal distribution [45]. The results indicated that there were significant effects of the subset of selected channels as well as the channel selection methods on the average errors across subjects (*p*-value = $4.69\times 10^{-8}$). Then, one-way ANOVA was applied to investigate the effect of channel selection methods with respect to the entire set of channels for each subset of channels. The results indicated that there are significant differences between the channel selection methods for subsets of three and one selected channels per muscle (*p*-value = 0.043 and F−crit(2.61)<F (2.72) and *p*-value = 0.0065 and F−crit(2.61)<F (4.11), respectively). However, no significant effect was found between PCA${}_{time}$, PCA${}_{freq}$, and PCR when comparing force estimation results for the seven-channel HD-EMG versus the two-channel per muscle subset (*p*-value = 0.49). To explore where the significant differences are, pairwise comparisons were done using the t-test for the subset of three channels, which resulted in improvements in force estimation for PCA${}_{freq}$ and PCR methods. The t-test results are shown in Table 2 for a subset of three channels. For this analysis, the Bonferroni correction method was applied at the 95% confidence level.

It is illustrated in Table 2 that there are significant effects when PCA${}_{freq}$ and PCR were employed for three-channel per muscle subset selection. However, there is no significant effect when PCA${}_{time}$ was used to select a three-channel per muscle subset versus the full set of HD signals.

We did not perform t-test analyses on single-channel subsets, since no improvement in force estimation was observed. When selecting one channel, the PCR method shows the closest performance to the full-set, followed by PCA${}_{freq}$ and PCA${}_{time}$. However, in single-channel selection, PCA${}_{time}$ and PCA${}_{freq}$ showed very high standard errors, indicating low consistency in force estimation. Evidently, while selection of only one channel provides a dimensionality reduction of 85.71%, it reduces our force estimation accuracy.

**Figure 2:**
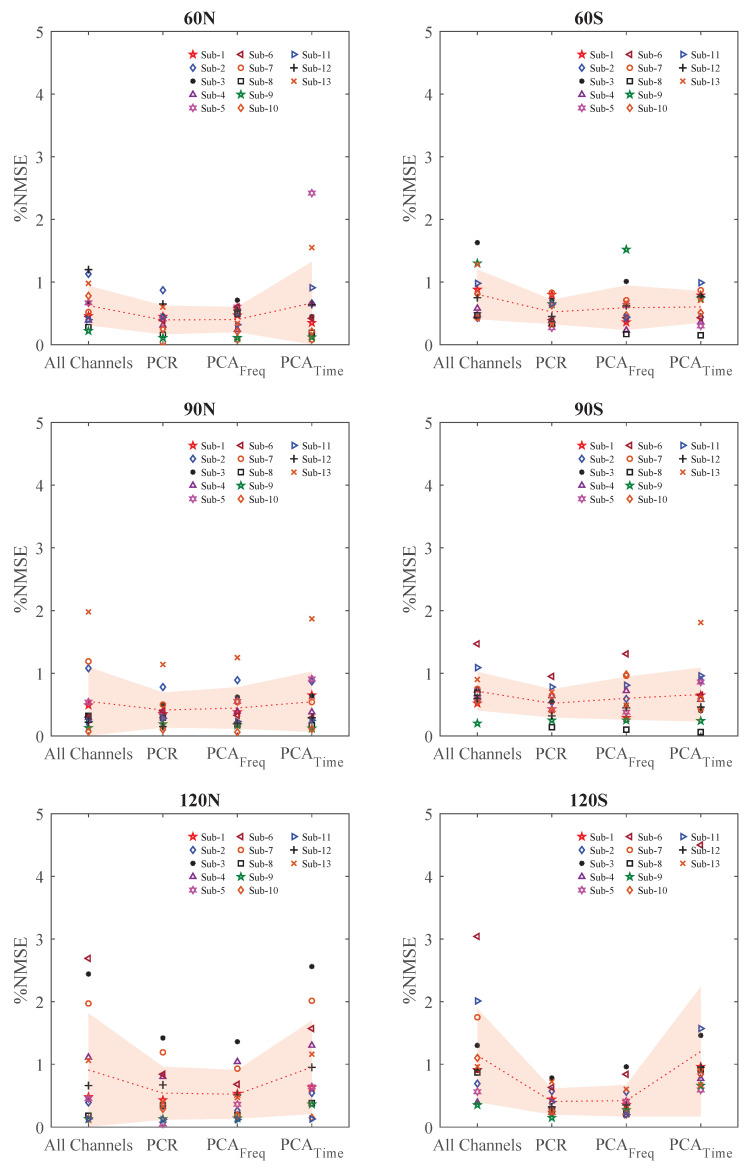
%NMSE values for 13 subjects, when a subset of 3 channels per muscle were selected by using power-correlation ratio (PCR), principal component analysis in the frequency domain (PCA${}_{freq}$), and PCA in the time domain (PCA${}_{time}$) in comparison with the full set of HD-EMG channels are shown.

Although there were no significant differences between the different approaches, for a subset of two channels, PCA${}_{time}$ still increased the average and standard deviation of the error in the estimated force, while PCA${}_{freq}$ and PCR reduced the error, achieving improvements. Moreover, the standard errors of the errors were considerably reduced using these two methods when two channels were selected. Finally, when a subset of three channels was selected, PCA${}_{time}$ further deteriorated the performance of the force-estimation process, while PCA${}_{freq}$ and PCR, averaged across all conditions, enhanced the results by almost 30%, and the standard errors were reduced. Figure 2 shows a detailed comparison of the %NMSE values obtained for the three-channel subsets selected for each subject in comparison with the all-channels case. The %NMSE values shown for each subject are averaged over the three force levels recorded. For almost all subjects, either the PCR or PCA${}_{freq}$ methods resulted in better performance than using all seven differential channels, and the average %NMSE values are lower than those for channel selection using PCA${}_{time}$.

These results indicate that while utilizing seven channels per muscle for force estimation yields relatively good results, there is redundant information among the channels, which is discarded through our channel selection techniques. On the other hand, it is evident that a single EMG channel does not contain enough information to allow for accurate force estimation.

**Figure 3:**
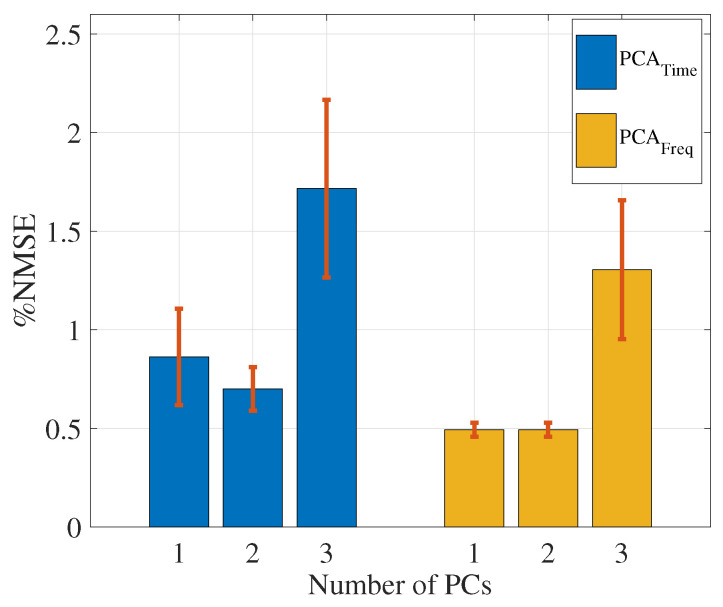
The means and standard errors of %NMSE for the estimated force are presented when the first, second, and third PCs were utilized for channel selection in the time and frequency domains.

Our proposed PCA technique (in both time and frequency domains) selects the channels with the largest contribution to the first PC. PCA in the time domain has been widely used as a common method to reduce the redundancy in data. However, we proposed the novel idea of using PCA in the frequency domain to select a subset of channels with the highest variance, where our results indicated that applying PCA in the frequency domain to select channels has the potential to improve the force estimation accuracy, while PCA in the time domain does not. To further explore the use of PCA for channel selection, we also examined voting for the selected channels based on the contributions of the PCs to the first two and first three PCs. The mean and SE values for each experimental condition are shown in Table 3, where the results suggest that considering more PCs does not improve the force modeling performance. As more PCs are selected in the time and frequency domains, the error is increased for each experimental condition. Figure 3 illustrates the average results across the different experimental conditions when the top one, two, and three PCs are considered.

**Figure 4:**
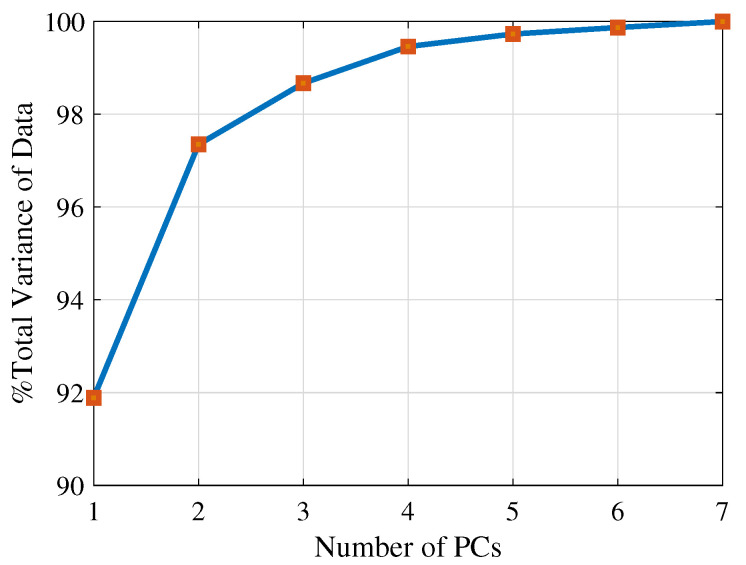
The percentage of the total variance explained by each PC for the long head of the biceps of one subject at $60^{\circ }$ joint angle and neutral posture.

These results were statistically investigated using single-factor ANOVA. The ANOVA results suggest that there are significant effects for using different numbers of PCs to select three channels for both PCA${}_{time}$ (*p* = 0.04) and PCA${}_{freq}$ (*p* = 0.0059). Next, a t-test was applied for pairwise comparison. The PCA methods using one and two PCs were not statistically different in either domain. When comparing the usage of one PC versus three PCs for channel selection, *p* = 0.08 for PCA${}_{time}$ and *p* = 0.019 for PCA${}_{freq}$; *p*= 0.021 for PCA${}_{time}$ and *p* = 0.019 for PCA${}_{freq}$ when comparing the usage of two PCs versus three PCs. However, considering the Bonferroni correction, there was no significant effect for using more than one PC for channel selection on force estimation accuracy in either the time or frequency domain. Therefore, using the first PC is sufficient to select channels to improve the force estimation accuracy because the first PC represents the maximum variation in the data. In Figure 4, the percentage of the total variance explained by each PC for the long head of the biceps brachii of one subject at $60^{\circ }$ joint angle, neutral posture, is shown. This figure shows that the first PC has 91.89% of the total variance, while the other components represent 5.46%, 1.32%, 0.79%, 0.27%, 0.14%, and 0.13%, respectively. Therefore, there is no need to use more than one PC to select a subset of channels, since the first PC contains the maximum information.

### 3.1. Evaluation and Comparison

Staudenmann et al. [24] used PCA on EMG signals, recorded using HD electrodes from the triceps brachii muscle during elbow extension, in order to improve EMG-based force estimation. They summed the PCs, and their evaluation criterion was the RMSD between the predicted and measured force values. They found that PCA reduced RMSD by approximately 12% for optimally aligned multiple bipolar electrodes. In our work, we did not use PCA to rebuild the EMG signal; instead, PCA was used to find the channels with the highest contribution (higher coefficient) in the PC space, indicating maximum variance of the signal. In addition, we achieved improvements of 29.92% and 29.17% NMSE compared to the seven-channel per muscle configuration using PCR and PCA${}_{freq}$ methods, respectively.

HD-EMG recordings were used for force estimation using an ensemble learning technique coupled with the FOS algorithm as an outlier detection method [10]. Four HD spatial configurations were considered. The lowest error was obtained in a configuration where all the bipolar channels (44 channels) were used for force estimation (mean %NMSE =2.43). In configurations where the number of channels was reduced (11 and seven channels), %NMSE =2.79 and %NMSE =2.99 were obtained, respectively. In this study, smaller subsets of channels were selected, but no attempt was made to optimize the chosen subset of channels. In comparison, through a similar reduction of approximately 50% in the number of channels, our method has been able to reduce the %NMSE through channel selection with PCR and PCA${}_{freq}$. This indicates the importance of channel selection not only for reducing the dimensionality, but also to improve the force modeling performance.

In a relatively different application of channel selection compared to our study, Geng et al. [27] proposed the MCCSP to select the optimal subset of channels from HD-EMG recordings for motion classification. The classification accuracy was defined as the percentage of the number of correct classifications over the total number of classifications. Eighteen monopolar EMG channels were selected among the total 56 as the sufficient number of channels for accurate motion classification, achieving an average classification accuracy of 93.03% in comparison to an accuracy of 94.50% for the entire set. When using a bipolar configuration, the average classification accuracy was 95.58% for 18 selected channels compared to 98.17% for the complete set.

Our channel selection methods provide a simple, practical, and effective way of dealing with the high dimensionality of EMG data in order to find a subset of channels that contributes the most to force estimation. PCA${}_{freq}$ and our PCR channel selection methods improve the force estimation accuracy by 29.17% and 29.92%, respectively, while reducing the dimensionality by 57%. Since we use a subject-dependent force estimation technique in FOS, our PCR and PCA techniques also select a unique set of channels for each subject. Our investigations showed that while either channel 3 or 4 was frequently among the selected channels (which matches the SENIAM recommended location), as expected, different subsets of channels were selected for force estimation for different subjects, as each subject has a unique physiology. To the best of our knowledge, our method outperforms available similar works in the literature that have tackled this problem.

### 3.2. Limitations

In regards to our proposed techniques, PCR and PCA${}_{freq}$, we are aware that the selected channels may not necessarily be the global optimum subset. In fact, a brute force search may result in a subset that is different from our proposed methods, further decreasing the subset size while increasing the accuracy of the estimated force. Nonetheless, a search approach is extremely time consuming, as all the possible subsets of channels need to be evaluated, which amounts to $2^{n}-1$ possible permutations where *n* is the number of channels (in our case, n=7 per muscle).

Another limitation of our method is that, similarly to many other studies in the field, the proposed channel selection technique is user-dependent, including the FOS algorithm used for force estimation in our study. Naturally, such algorithms depend on parameters learned from the user prior to deployment. This is also the case for our proposed channel selection method, as it requires the power spectral density and correlation between channels from the user, which, in fact, can be measured together with the calibration/learning phase of the FOS force estimation technique. In addition, the selected subset of channels may, in fact, change with the choice of force estimation technique. For example, we have yet to explore whether our method would find the same subset if an artificial neural network classifier was used instead of FOS. The same uncertainty applies to the pre-processing steps. However, the pre-processing methods used in this paper are fairly standard and common when utilizing EMG.

A general challenge with HD electrodes is that, compared to regular single-channel electrodes, HD electrodes have a higher chance of facing artifacts in the recordings given the relatively large area of the electrode pads, which can lift from the skin or may bend during muscle flexion. In such cases, re-recording of the data is often necessary in order to obtain usable recordings for EMG-based force estimation.

Finally, the third limitation of our work is the fact that we have not evaluated the approach on larger numbers of EMG channels or for other types of contractions. While we expect our approach to generalize fairly well to larger sets and different contractions (for example, two-dimensional HD-EMG, which has a greater number of channels, or isotonic/dynamic contractions), we have yet to study such scenarios.

## 4. Conclusions and Future Work

In this study, linear electrode arrays were used to record EMG signals during isometric contractions over three elbow flexor muscle locations while simultaneously recording the generated force. The recorded EMG data were first pre-processed, and the force induced at the wrist was estimated using FOS. Two techniques, one based on PCA in the frequency domain and a second, novel index called the power-correlation ratio (PCR), were used to select a subset of channels for force estimation. PCA${}_{freq}$ and PCR improved the force estimation accuracy by 29.17% and 29.92% while reducing the dimensionality by 57%.

Both proposed methods, PCA${}_{freq}$ and PCR, achieved lower errors for force modeling in comparison with the full set and the baseline, PCA${}_{time}$, when a subset of three channels per muscle was selected. Although the accuracy of the PCR is slightly better than that of PCA${}_{freq}$ for most of the subjects and experimental conditions, as shown in Figure 2, they are not statistically different. However, the performance of PCR for the subset of one and two channels is better than that of PCA${}_{freq}$, and is much closer to the full set. This indicates that the PCR is better approach than PCA${}_{freq}$ when dimensionality reduction is more important, since compromising the accuracy is lower with PCR than PCA${}_{freq}$ if fewer number of channels will be selected. In addition, in this paper, we showed that using the first PC is sufficient to select channels to improve the force estimation accuracy, though this might need more investigation if the application is changed. Therefore, more analysis is needed to implement PCA${}_{freq}$ compared to the PCR.

For future work, we will explore machine learning and deep learning techniques for channel selection by training supervised models. Additionally, we will investigate the estimation performance of the FOS model over time. Moreover, in addition to FOS, other force estimation methods such as machine learning approaches, for example, using neural networks, will be used, and our method will be evaluated. Furthermore, we will explore different types of muscle contractions, such as dynamic contraction. Finally, we believe that by incorporating information regarding the dynamics of joints, for example, through wearable inertial sensors, more accurate force estimation and channel selection may be achieved.

## Figures and Tables

**Table 1:** Mean and standard error (SE) values of the normalized mean square error (%NMSE) for the different channel selection methods used are given for the different experimental conditions. The numbers of channels selected per muscle are shown. The elbow joint angles are 60, 90, and 120, and N and S represent neutral and supinated posture.

Method	Channel	60N	60S	90N	90S	120N	120S
**PCA*_time_***	1	1.67 ± 0.53	1.98 ± 0.86	0.75 ± 0.21	**0.84** ± **0.28**	1.04 ± 0.27	2.44 ± 1.14
**PCA*_time_***	2	0.52 ± 0.12	1.32 ± 0.71	**0.57** ± **0.09**	0.72 ± 0.23	1.02 ± 0.33	1.78 ± 0.18
**PCA*_time_***	3	0.64 ± 0.18	0.60 ± 0.09	0.55 ± 0.08	0.66 ± 0.1	0.91 ± 0.44	1.18 ± 0.5
**PCA*_freq_***	1	1.67 ± 0.7	**0.90** ± **0.21**	1.40 ± 0.5	1.19 ± 0.3	0.96 ± 0.27	1.97 ± 0.81
**PCA*_freq_***	2	**0.51** ± **0.11**	0.65 ± 0.13	0.70 ± 0.15	**0.59** ± **0.1**	**0.62** ± **0.16**	**0.63** ± **0.18**
**PCA*_freq_***	3	**0.40** ± **0.05**	0.59 ± 0.12	0.45 ± 0.08	0.60 ± 0.09	**0.52** ± **0.09**	0.42 ± 0.06
**PCR**	1	**0.97** ± **0.23**	1.08 ± 0.52	**0.65** ± **0.1**	1.20 ± 0.33	**0.93** ± **0.24**	**0.92** ± **0.24**
**PCR**	2	**0.51** ± **0.09**	**0.54** ± **0.11**	0.62 ± 0.11	0.63 ± 0.11	**0.62** ± **0.15**	0.89 ± 0.3
**PCR**	3	0.41 ± 0.07	**0.55** ± **0.08**	**0.41** ± **0.07**	**0.54** ± **0.06**	0.54 ± 0.1	**0.41** ± **0.05**
**All Channels**	7	0.54 ± 0.076	0.81 ± 0.26	0.55 ± 0.11	0.70 ± 0.14	0.89 ± 0.27	1.01 ± 0.34

**Table 2:** T-test results for pairwise comparison of different methods for a subset of three channels per muscle.

Comparison	*p*-Value	*t*-Stat	*t*-Critical
All Channels, PCR	**0.0049**	2.84	1.97
All Channels, PCA${}_{freq}$	**0.008**	2.6	1.97
All Channels, PCA${}_{time}$	0.76	0.31	1.97
PCR, PCA${}_{freq}$	0.18	1.32	1.97
PCR, PCA${}_{time}$	0.028	2.2	1.97
PCA${}_{freq}$, PCA${}_{time}$	0.05	1.98	1.97

**Table 3:** Mean and SE values for the first, second, and third principal components (PCs) used for channel selection in the time and frequency domains for each experimental condition are shown.

	PCA_*freq*_			PCA_*time*_		
	**1 PC**	**2 PC**	**3 PC**	**1 PC**	**2 PC**	**3 PC**
**60N**	0.40 ± 0.05	0.40 ± 0.05	1.53 ± 1.02	0.64 ± 0.18	0.52 ± 0.09	0.64 ± 0.16
**60S**	0.59 ± 0.12	0.59 ± 0.12	0.55 ± 0.11	0.56 ± 0.09	0.53 ± 0.09	4.21 ± 2.23
**90N**	0.45 ± 0.08	0.45 ± 0.08	0.45 ± 0.08	0.52 ± 0.08	0.52 ± 0.09	0.75 ± 0.2
**90S**	0.60 ± 0.09	0.60 ± 0.09	2.05 ± 1.09	0.66 ± 0.1	0.66 ± 0.11	2.85 ± 1.43
**120N**	0.52 ± 0.1	0.52 ± 0.1	1.58 ± 0.76	0.89 ± 0.44	0.92 ± 0.44	1.58 ± 0.76
**120S**	0.42 ± 0.07	0.42 ± 0.07	1.62 ± 1.16	1.08 ± 0.5	1.02 ± 0.43	0.77 ± 0.17
